# Imputing the Number of Responders from the Mean and Standard Deviation of CGI-Improvement in Clinical Trials Investigating Medications for Autism Spectrum Disorder

**DOI:** 10.3390/brainsci11070908

**Published:** 2021-07-09

**Authors:** Spyridon Siafis, Alessandro Rodolico, Oğulcan Çıray, Declan G. Murphy, Mara Parellada, Celso Arango, Stefan Leucht

**Affiliations:** 1 Department of Psychiatry and Psychotherapy, School of Medicine, Technical University of Munich, 81675 Munich, Germany; stefan.leucht@tum.de; 2 Department of Experimental and Clinical Medicine, Psychiatric Clinic University Hospital ‘Gaspare Rodolico’, University of Catania, 95125 Catania, Italy; alessandro.rodolico@me.com; 3 Department of Child and Adolescent Psychiatry, Mardin State Hospital, 47100 Artuklu, Mardin, Turkey; remziogulcanciray@gmail.com; 4 Department of Forensic and Neurodevelopmental Sciences, Institute of Psychiatry, Psychology & Neuroscience, King’s College London, London WC2R 2LS, UK; declan.murphy@kcl.ac.uk; 5 Department of Child and Adolescent Psychiatry, Institute of Psychiatry and Mental Health, Hospital General Universitario Gregorio Marañón, 28003 Madrid, Spain; parellada@hggm.es (M.P.); carango@hggm.es (C.A.); 6 School of Medicine, Universidad Complutense, 28040 Madrid, Spain

**Keywords:** response, meta-analysis, continuous outcomes, dichotomous outcomes

## Abstract

Introduction: Response to treatment, according to Clinical Global Impression-Improvement (CGI-I) scale, is an easily interpretable outcome in clinical trials of autism spectrum disorder (ASD). Yet, the CGI-I rating is sometimes reported as a continuous outcome, and converting it to dichotomous would allow meta-analysis to incorporate more evidence. Methods: Clinical trials investigating medications for ASD and presenting both dichotomous and continuous CGI-I data were included. The number of patients with at least much improvement (CGI-I ≤ 2) were imputed from the CGI-I scale, assuming an underlying normal distribution of a latent continuous score using a primary threshold θ = 2.5 instead of θ = 2, which is the original cut-off in the CGI-I scale. The original and imputed values were used to calculate responder rates and odds ratios. The performance of the imputation method was investigated with a concordance correlation coefficient (CCC), linear regression, Bland–Altman plots, and subgroup differences of summary estimates obtained from random-effects meta-analysis. Results: Data from 27 studies, 58 arms, and 1428 participants were used. The imputation method using the primary threshold (θ = 2.5) had good performance for the responder rates (CCC = 0.93 95% confidence intervals [0.86, 0.96]; β of linear regression = 1.04 [0.95, 1.13]; bias and limits of agreements = 4.32% [−8.1%, 16.74%]; no subgroup differences χ^2^ = 1.24, *p*-value = 0.266) and odds ratios (CCC = 0.91 [0.86, 0.96]; β = 0.96 [0.78, 1.14]; bias = 0.09 [−0.87, 1.04]; χ^2^ = 0.02, *p*-value = 0.894). The imputation method had poorer performance when the secondary threshold (θ = 2) was used. Discussion: Assuming a normal distribution of the CGI-I scale, the number of responders could be imputed from the mean and standard deviation and used in meta-analysis. Due to the wide limits of agreement of the imputation method, sensitivity analysis excluding studies with imputed values should be performed.

## 1. Introduction

There is still no approved medication for the core symptoms of autism spectrum disorder (ASD) (i.e., social communication difficulties and repetitive restricted behaviors [1]), yet a large number of medications are being investigated in an increasing number of randomized controlled trials (RCTs), with this number increasing sharply after 2008 [2]. Many of these trials are pilot trials with small sample sizes and cannot provide definite answers, and given their increasing number, there is an ongoing need to comprehensively synthesize their evidence [2].

However, the lack of agreement on the selection of outcome measures for the core symptoms in clinical trials precludes the synthesis of evidence [3,4,5]. The available scales are, at best, “appropriate with conditions“ [3,4], and given the lack of a “gold standard”, the Clinical Global Impression scales (CGI-Severity and CGI-Improvement) [6,7] have been widely used in clinical trials of ASD [8,9] not only as important secondary outcomes, but also as the primary outcome [10]. CGI-Severity (CGI-S) is a seven-point scale used by clinicians to assess the current severity of illness, ranging from one (“normal, not at all ill”) to seven (“among the most extremely ill patients”) and usually measured at the trial’s baseline and endpoint. CGI-Improvement (CGI-I) is a seven-point scale used by clinicians to measure global response compared to the baseline, ranging from one (“very much improved”) to seven (“very much worse”). A clinically important response is frequently defined as at least much improvement (i.e., a number of participants with a CGI-I score of one or two) [11].

In addition, a comprehensive synthesis of evidence would require the combination of all available studies; however, some of them may present the CGI-I as a continuous outcome (i.e., with a mean and standard deviation). The conversion of continuous outcomes to dichotomous ones would allow the combination all available data across studies. Imputation methods of the number of responders from the means and standard deviations have been validated with depression [12] and schizophrenia scales [13]. The appropriateness of these methods might be questioned with the CGI-I, given the limited number of points of the CGI, as well as in ASD, given its heterogeneity and the small sample sizes of clinical trials (only 8.7% of RCTs included more than 100 participants [2]). Therefore, our aim was to validate the imputation of the responder rates from the means and standard deviations of the CGI-I in ASD trials. We compared the responder rates and odds ratios calculated from the original and imputed numbers of participants with a clinically important response to treatment.

## 2. Methods

### 2.1. Dataset

This is a secondary analysis which uses part of the dataset from a systematic review and meta-analysis on pharmacological and dietary supplement interventions for ASD (PROSPERO ID: CRD42019125317) [14,15]. A comprehensive literature search, study selection, and data extraction by at least two independent reviewers were conducted (last update search on 31 August 2020). Response to treatment was investigated as a secondary outcome in the reviews, and the CGI-I was extracted as continuous and dichotomous outcomes. In this analysis, we used 27 studies with 58 arms and 1428 participants that provided data on (1) the means and standard deviations (SDs) of the CGI-I and (2) the number of responders defined at least as much improved in the CGI-I (CGI-I ≤ 2). Data from the endpoint of the studies were used (the minimum duration of treatment was set at seven days). The intention-to-treat (ITT) data were preferred, and when only completer data was available, we assumed that participants lost to the follow-up did not respond.

The cut-off of the least much improvement (CGI-I 1 or 2) was investigated, which represents a clinically important response [11] and is frequently reported in clinical trials [10]. The responder rates using the original or imputed number of responders were calculated in each arm. The odds ratios (ORs) were also calculated for each non-reference arm in a study, using as a reference the placebo arm of the study or another active treatment (in the case of non-placebo-controlled trials).

### 2.2. Imputation Method

**Figure 1:**
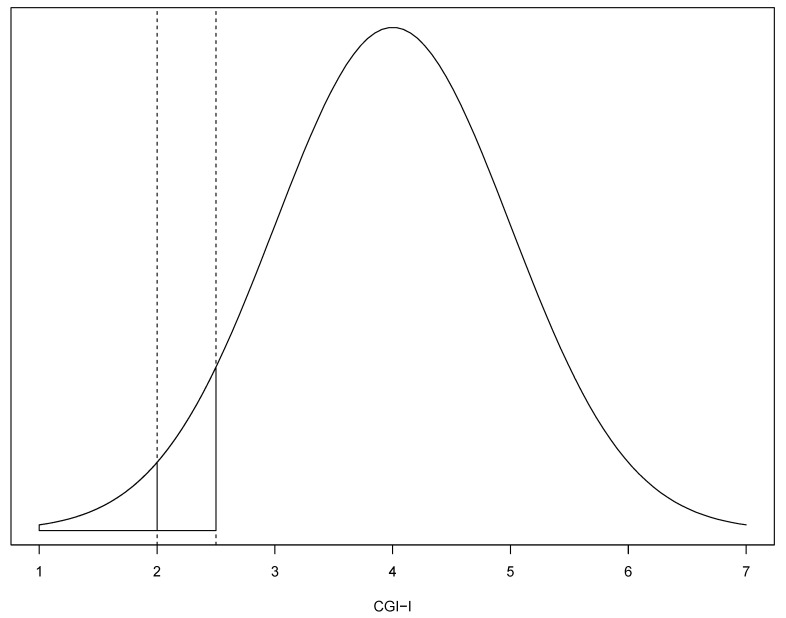
Underlying distribution of a latent CGI-I score, using an assumed normal distribution of the CGI-I, such as with μ = 4 and σ = 1. Under the assumption of a normal distribution, the probability (p) of at least much improvement (CGI-I = 2) could be calculated with Z-score = (θ−μ)/σ, where θ is a threshold of the response. As a primary threshold, we used θ = 2.5 for at least much improvement (CGI-I of 1 or 2, the blue and red shaded parts of the distribution), since it could be assumed that a patient with a score between 2 and 2.5 in the underlying latent continuous variable would have been classified as at least much improved. As a secondary threshold, we used θ = 2 (red shaded part of the distribution).

We used an imputation method validated with depression [12] and schizophrenia scales [13] which assumed a normal distribution of the scale (CGI-I in this analysis) given a mean (μ) and standard deviation (σ). The number of responders of a threshold (θ) in the CGI-I (i.e., participants with a CGI-I score ≤ θ) could be calculated using the total number of participants assessed (n) and the probability of the lower tail of the distribution (*p*) for Z-score = (θ − μ)/σ (Figure 1). Then, the number of responders was n * *p*.

According to the work of Furukawa et al. in 2005 [12], when the CGI-I was used, responders were imputed using the threshold of θ = 2 (at least “much improved”). However, the CGI-I is a seven-point Likert-type scale, and an underlying latent continuous variable could be assumed which could have had different thresholds of mapping the discrete responses [16]. Both the ordinal scale scores and the scores of the latent continuous variable would have the same μ and σ, but the threshold θ for the discrete responses (e.g., of at least “much improved”) would differ [16]. Therefore, we used a threshold of θ = 2.5 as the primary threshold to impute the number of responders (Figure 1), since a participant with a latent CGI-I continuous score ranging from 2 to 2.5 would have also been considered as at least “much improved”. In a secondary analysis, we used a secondary threshold of θ = 2 to impute responders from the assumed normal distribution of the ordinal scale.

We calculated the responder rates from the original and imputed numbers of responders using the randomized number of participants as the denominator. We also calculated the odds ratios (OR) between the experimental and control investigations (placebo or another active treatment). The natural logarithm of the ORs (lnOR) was used in the analysis.

### 2.3. Assessment of Performance of the Imputation Method

#### 2.3.1. Concordance Correlation Coefficient (CCC)

The agreement between the original and imputed responder rates and the lnORs were investigated with the concordance correlation coefficient (CCC) [17] and its 95% confidence intervals. The CCC ranged between −1 and 1 (perfect agreement).

#### 2.3.2. Predictive Accuracy and Linear Regression Model

Linear regression models were used to determine the predictive accuracy of the imputation method, and a good imputation method should have a slope (β) and R^2^ close to one and a low mean squared error (MSE).

#### 2.3.3. Limits of Agreement and Bland–Altman Analysis

The Bland–Altman method was used to investigate the limits of agreement of the bias (i.e., the difference between the original and imputed values) [18,19]. In the Bland–Altman plot, the difference of the original and imputed values is presented in the *y*-axis, and their average is in the *x*-axis. The distribution of the difference was inspected for normality, and a Shapiro–Wilk test was conducted. The limits of agreement were represented with 95% confidence intervals, considering acceptable the ones found in the validation of the method in schizophrenia scales [13], i.e., −0.7% 95% CI (−9.8%, 8.4%) for the difference of the original and imputed responder rates and 0.06 95% CI (−0.24, 0.35) for the difference of the original and imputed lnORs. To investigate if the bias was proportional to the mean, a linear regression model of the differences on their mean (using the natural logarithms for both the responder rates and odds ratios) was conducted [18].

### 2.4. Meta-Analysis

We compared the pooled estimates from the meta-analysis using the original and imputed values. The responder rates (logit transformed and back-transformed for presentation) [20] and odds ratios (natural logarithm and back-transformed for presentation) were pooled in a random-effects meta-analysis [21]. Subgroup analysis was conducted to investigate the differences of the pooled estimates from the meta-analysis using the original and the imputed values (primary and secondary thresholds).

Analysis was conducted in R v4.0.3 [22]. The CCC, linear regression, and Bland–Altman limits were calculated with base R and epiR v2.0.17 [23]. The effect sizes and meta-analysis were calculated with metafor v2.4−0 [24] and meta v4.15−1 [25]. The data cleaning and graphs were completed using packages of tidyverse v13.0 [26]. The statistical threshold was set at two-sided alpha 5%.

## 3. Results

**Figure 2:**
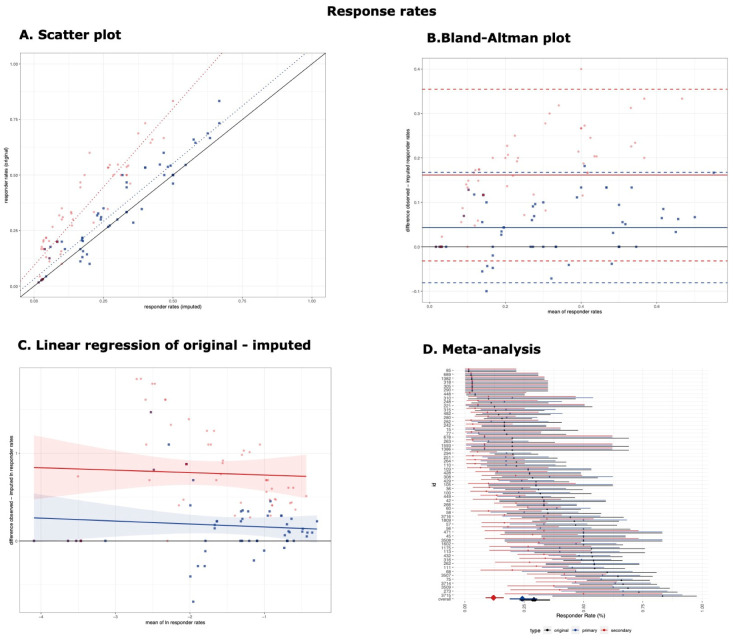
**Response rates.** (**A**) Scatter plot of response rates. Scatter plot of the comparison between original and imputed response rates (blue for the primary threshold and red for the secondary threshold). The black solid line represents the line of perfect correspondence. Blue and red dotted lines represent the linear regression model for the primary and secondary threshold. (**B**) Bland-Altman plot of response rates. The black solid line represents the optimal difference between original and imputed responder rates. The solid blue and red lines represent the median difference of the primary and secondary threshold, and the dashed blue and red dotted lines represent their 95% confidence intervals, corresponding to the limits of agreement. (**C**) Linear regression of original minus imputed ln responder rates. Linear regression of the difference between original and imputed natural logarithms of responder rates to their mean. Regression lines and its 95% confidence intervals are presented for the primary threshold (blue) and the secondary threshold (red). (**D**) Meta-analysis. Meta-analysis of responder rates using original values (black), imputed using the primary threshold (blue) and secondary threshold (red). Effect sizes with their 95% confidence intervals are presented with circles and error bars for individual arms and with diamonds and error bars for the pooled estimates.

**Figure 3:**
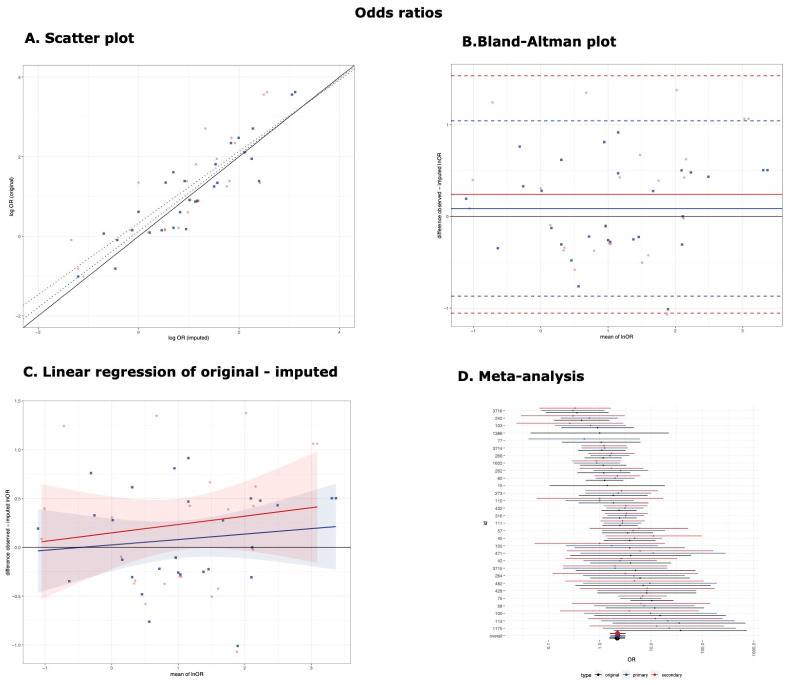
**Odds ratios.** (**A**) Scatter plot of lnORs. Scatter plot of the comparison between original and imputed lnORs (blue for the primary threshold and red for the secondary threshold). The black solid line represents the line of perfect correspondence. Blue and red dotted lines represent the linear regression model for the primary and secondary threshold. (**B**) Bland-Altman plot of lnORs. The black solid line represents the optimal difference between original and imputed lnORs. The solid blue and red lines represent the mean difference of the primary and secondary threshold, and the dashed blue and red dotted lines represent their 95% confidence interval of the difference, corresponding to the limits of agreement. (**C**) Linear regression of original minus imputed lnOR. Linear regression of the difference between original and imputed natural logarithms of odds ratios to their mean. Regression lines and its 95% confidence intervals are presented for the primary threshold (blue) and the secondary threshold (red). (**D**) Meta-analysis of odds ratios. Meta-analysis of odds ratios using original values (black), imputed using the primary threshold (blue) and secondary threshold (red). Effect sizes with their 95% confidence intervals are presented with circles and error bars for individual arms and with diamonds and error bars for the pooled estimates.

The results of the CCC, linear regression, and Bland–Altman analysis are presented in Table 1 and Figure 2 (responder rates) and Figure 3 (odds ratios).

### 3.1. Responder Rates

The responder rates derived from the imputed values using the primary threshold (θ = 2.5) were in good agreement with the original values (CCC 0.93, 95% confidence interval [0.89, 0.96]), and the imputation method had good predictive accuracy (β = 1.04 [0.95, 1.13], R^2^ = 90.86%, MSE = 0.063) (Figure 2A, blue). The difference between the original and imputed values (normally distributed, Figure S1) was, on average, 4.32% with 95% confidence intervals [−8.1%, 16.74%] (Figure 2B, blue), and it was not proportional to the mean when natural logarithms were used (β = −0.034 [−0.135, 0.068]) (Figure 2C, blue).

On the other hand, the imputation method had poorer performance when the secondary threshold was used (θ = 2), with poor agreement (CCC = 0.59 [0.48, 0.69]) and predictive accuracy (β = 1.41 [1.26, 1.57], R^2^ = 85.01%, MSE = 0.0813). This would mean that the original responder rates of 20% would correspond, on average, to imputed responder rates of 14.2% and from 50% to 35.46% (1.41 times higher) (Figure 2A, red). The difference between the original and imputed values (normally distributed, Figure S2) was larger on average (16.15% [−3.18%, 35.47%]) (Figure 2B, red) and not proportional to the mean when natural logarithms were used (β = −0.034 [−0.135, 0.068]) (Figure 2C, red). In comparison with the schizophrenia scales (bias −0.7% [−9.8%, 8.4%]) [13], the bias was larger and the limits of agreements were wider.

The summary estimates obtained from the meta-analysis of the imputed values using the secondary threshold (12.1% [8.8%, 16.4%]) were smaller than those obtained from the imputed values using the primary threshold (24.3% [19%, 30.4%]) or the original values (29.1% [23.2%, 35.8%]) (χ^2^ = 22.22, *p*-value < 0.001) (Figure 2D). This was reflected in the post hoc two-by-two comparisons that found the summary estimates obtained from the imputed values using the secondary threshold were smaller than those using the primary threshold (χ^2^ = 12.29, *p*-value < 0.001) or original values (χ^2^ = 21, *p*-value < 0.001), while there was no difference between the latter two (χ^2^ = 1.24, *p*-value = 0.266).

### 3.2. Odds Ratios

When the primary threshold was used (θ = 2.5), the imputed natural logarithm of the odds ratios was in good agreement with the original values (CCC 0.91, 95% confidence interval [0.81, 0.95]), and the imputation method had good predictive accuracy (β = 0.96 [0.78, 1.14], R^2^ = 82.03%, MSE = 0.495) (Figure 3A, blue). The difference between the original and imputed values (normally distributed, Figure S3) was, on average, 0.09 with 95% confidence intervals [−0.87, 1.04] (Figure 3B, blue). This would mean that the original odds ratios were, on average, 1.1 (=e^0.09^) times larger than the imputed values (95% CI [0.42, 2.83]). The differences were not proportional to the mean (β = 0.06 [−0.120, 0.231]) (Figure 3C, blue).

The imputation method using the secondary threshold (θ = 2) had poorer performance, with a CCC of 0.81 [0.63, 0.91]) and predictive accuracy of β = 0.90 [0.65, 1.15], R^2^ = 67.85%, MSE = 0.664 (Figure 3A, red). The difference between the original and imputed values (normally distributed, Figure S4) was, on average, 0.24 [−1.05, 1.53] (Figure 2B, red), meaning that the original odds ratios were, on average, 1.27 (=e^0.24^) times larger than the imputed values (95% [0.35, 4.62]). The differences were not proportional to the mean (β = 0.086 [−0.164, 0.334]) (Figure 3C, red). For both thresholds, the average bias was similar, yet the limits of agreement were considerably wider than those found in the schizophrenia scales (0.06 [−0.24, 0.35]) [13].

Nevertheless, no subgroup differences were found in the pooled estimates obtained from the meta-analysis, regardless of whether the original values (number of observations k = 30, 2.20 [1.56, 3.09]) or the imputed values using the primary (k = 28, 2.27 [1.64, 3.14]) or secondary threshold (k = 27, 2.23 [1.60, 3.11]) were used (χ^2^ = 0.02, *p*-value = 0.991) (Figure 3D). No subgroup differences were found in the post hoc two-by-two comparisons (i.e., original versus imputed using the primary threshold (χ^2^ = 0.02, *p*-value = 0.894), original versus secondary threshold (χ^2^ < 0.00, *p*-value = 0.949), and primary versus secondary threshold (χ^2^ < 0.00, *p*-value = 0.945)). It should be noted that the odds ratios were not calculated in the case of double zeros (i.e., no responder in the experimental or control interventions). Therefore, some original observations were not paired with the imputed observations in these meta-analyses (2 out of 30 for the primary threshold and 3 out of 30 for the secondary threshold).

## 4. Discussion

In this analysis, we applied an imputation method previously validated mainly with depression [12] and schizophrenia scales [13] to estimate the number of responders from the means and standard deviations of the CGI-I in ASD. We further replicated the quite satisfactory performance of the imputation method, suggesting that the number of responders could be imputed from the CGI-I, and they could be used in the meta-analysis of the responder rates and odds ratios. Our findings also suggest that, since the imputation method assumed a normal distribution of the seven-point Likert-type CGI-I scale, an underlying latent continuous variable could be considered, and a higher threshold than the original could be used in the imputation method for better performance, such as with participants that were at least much improved (CGI-I ≤ 2), which would have had a score in the latent continuous variable ≤2.5. In a previous study validating the method in depression [12], the number of responders was imputed in a subset of studies from the CGI-I using the original threshold of “at least much improvement” (θ = 2), yet the specific performance on the CGI-I was not evaluated. Nevertheless, differences between the primary and secondary thresholds were less striking when the odds ratios were used in comparison with the response rates, since relative indices like odds ratios seem to remain constant across different thresholds and control event rates [27].

Our analysis would facilitate synthesis of evidence in ASD by allowing the conversion of the means and standard deviations of the CGI-I to number of responders and subsequent meta-analysis to incorporate all available data. There is still no consensus on the selection of the outcome measures of symptom change in ASD, so diverse scales that assess different symptom domains (e.g., social communication difficulties, repetitive behaviors, and problem behaviors) have been used across trials. The majority of them are not specifically designed to measure treatment response, and only a few have been used in more than 5% of clinical trials [9]. On the other hand, the CGI-I is recommended for use in clinical trials irrespective of their objective and clinical context in order to measure treatment response while incorporating all behavior symptom domains [8,9]. Therefore, pooled estimates derived from the number of responders according to the CGI-I might be more clinically interpretable than those from the standardized mean differences (SMDs) of diverse scales [28].

This analysis has certain limitations. First, there were considerable data for the responder rates (27 studies and 58 arms), yet the data points on the odds ratios were about half the amount (because a reference should be used in each study), also resulting in wider limits of agreements. Second, we focused on the clinically important response using the cut-off of “at least much improvement”, or CGI-I ≤ 2. Therefore, the imputation method was not directly validated for the other cut-offs, such as “at least minimal improvement”, or CGI-I ≤ 3. Third, our data were derived from clinical trials investigating pharmacological and dietary supplement interventions for ASD. Therefore, generalizability to psychosocial interventions or other fields of medicine should be further examined. Fourth, the imputation method assumes a normal distribution, yet scores from a Likert-type scale like the CGI-I might be frequently skewed. Indeed, potential skewness was suggested in 45% of the arms (when mean − 1 < 2 * SD), and there was strong evidence of skewness in 5% of the arms (when mean − 1 < SD) (Figure S5) [29]. Nevertheless, the performance of the imputation method was surprisingly satisfactory. Fifth, other methods to convert continuous to dichotomous effect sizes (e.g., from SMD to OR) have been proposed [30] and were not evaluated here, yet the method in this manuscript allows for the estimation of the number of responders that could be used in meta-analysis of both the proportions (such as single-group meta-analysis of responder rates) and relative effects (such as odds ratios or relative risks).

In conclusion, the number of responders could be imputed when given a mean and standard deviation of CGI-I. The imputation method had better performance when an underlying latent continuous variable was considered and an appropriate threshold was used (θ = 2.5 and not 2 for “at least much improvement”). The imputed number of responders could be used in meta-analysis of the responder rates and odds ratios. Given the wide limits of agreement between the original and imputed values, the robustness of the results of the main analysis should be investigated in a sensitivity analysis by excluding effect sizes derived from the imputed number of responders, as has been suggested previously [13].

## Figures and Tables

**Table 1:** CCC and regression of response rates.

		Agreement	Predictive Accuracy			Bias	
	Number of Observations (k)	CCC (95% CI)	β (95% CI) of Original (Y) and Imputed (X)	R^2^ (%)	MSE	Bias and 95% Limits of Agreement	β (95% CI) of Difference (Y) and Mean (X)
**Responder Rates (Original 58 Observations)**							
**Primary Threshold**	58	0.93 (0.89–0.96)	1.04 (0.95, 1.13)	90.86	0.063	4.32% (−8.1%, 16.74%)	−0.034 (−0.135, 0.068) *
**Secondary Threshold**	58	0.59 (0.48−0.69)	1.41 (1.26, 1.57)	85.01	0.0813	16.15% (−3.18%, 35.47%)	−0.028 (−0.177, 0.121) *
**Log OR (Original 30 Observations)**							
**Primary Threshold**	28	0.91 (0.81, 0.95)	0.96 (0.78, 1.14)	82.03%	0.495	0.09 (−0.87, 1.04)	0.06 (−0.120, 0.231)
**Secondary Threshold**	27	0.81 (0.63, 0.91)	0.90 (0.65, 1.15)	67.85%	0.664	0.24 (−1.05, 1.53)	0.086 (−0.164, 0.334)

* Natural logarithmic transformation of the responder rates. The dependent and independent variables of linear regressions are indicated with (Y) and (X), respectively.

## Data Availability

The data presented in this study are available on request from the corresponding author.

## Supplementary Materials

The following are available online at https://www.mdpi.com/article/10.3390/brainsci11070908/s1, Figure S1: Histogram and QQ plot of original-imputed responder rates (primary threshold), Figure S2: Histogram and QQ plot of original-imputed responder rates (secondary threshold), Figure S3: Histogram and QQ plot of original-imputed lnOR (primary threshold), Figure S4: Histogram and QQ plot of original-imputed lnOR (secondary threshold), Figure S5: Investigation of skewness of CGI-I scores.
