# Supplementary material for: Imputing the Number of Responders from the Mean and Standard Deviation of CGI-Improvement in Clinical Trials Investigating Medications for Autism Spectrum Disorder
Source: Brain Sci. 2021 Jul 9;11(7):908. doi: 10.3390/brainsci11070908 (PMC8305379; doi:10.3390/brainsci11070908)
Supplement: Supplementary file 1 [file brainsci-11-00908-s001.zip › brainsci-1272885-supplementary.pdf]

Supplementary content

Figure-S1 Histogram and QQ plot of original-imputed responder rates (primary threshold)

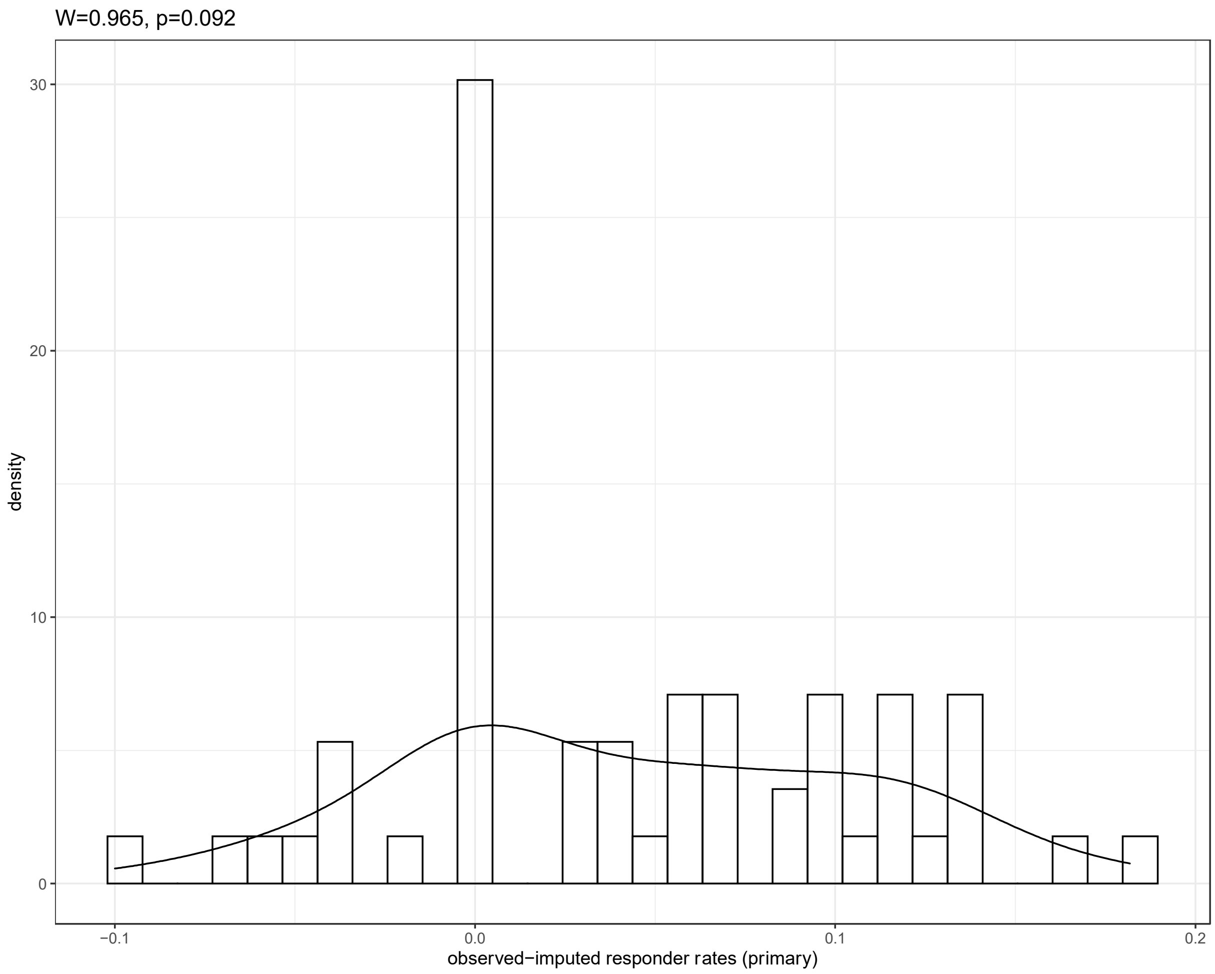

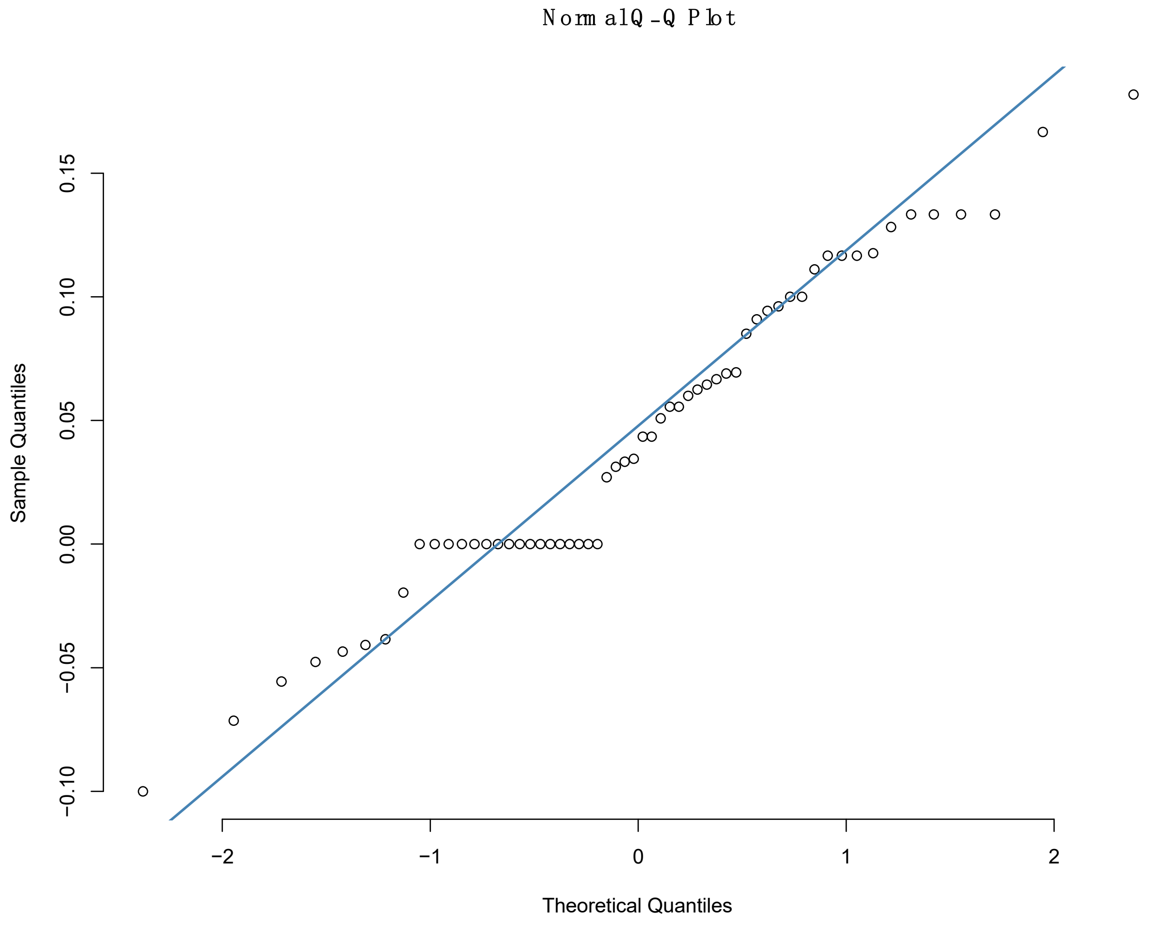

Figure-S2 Histogram and QQ plot of original-imputed responder rates (secondary threshold)

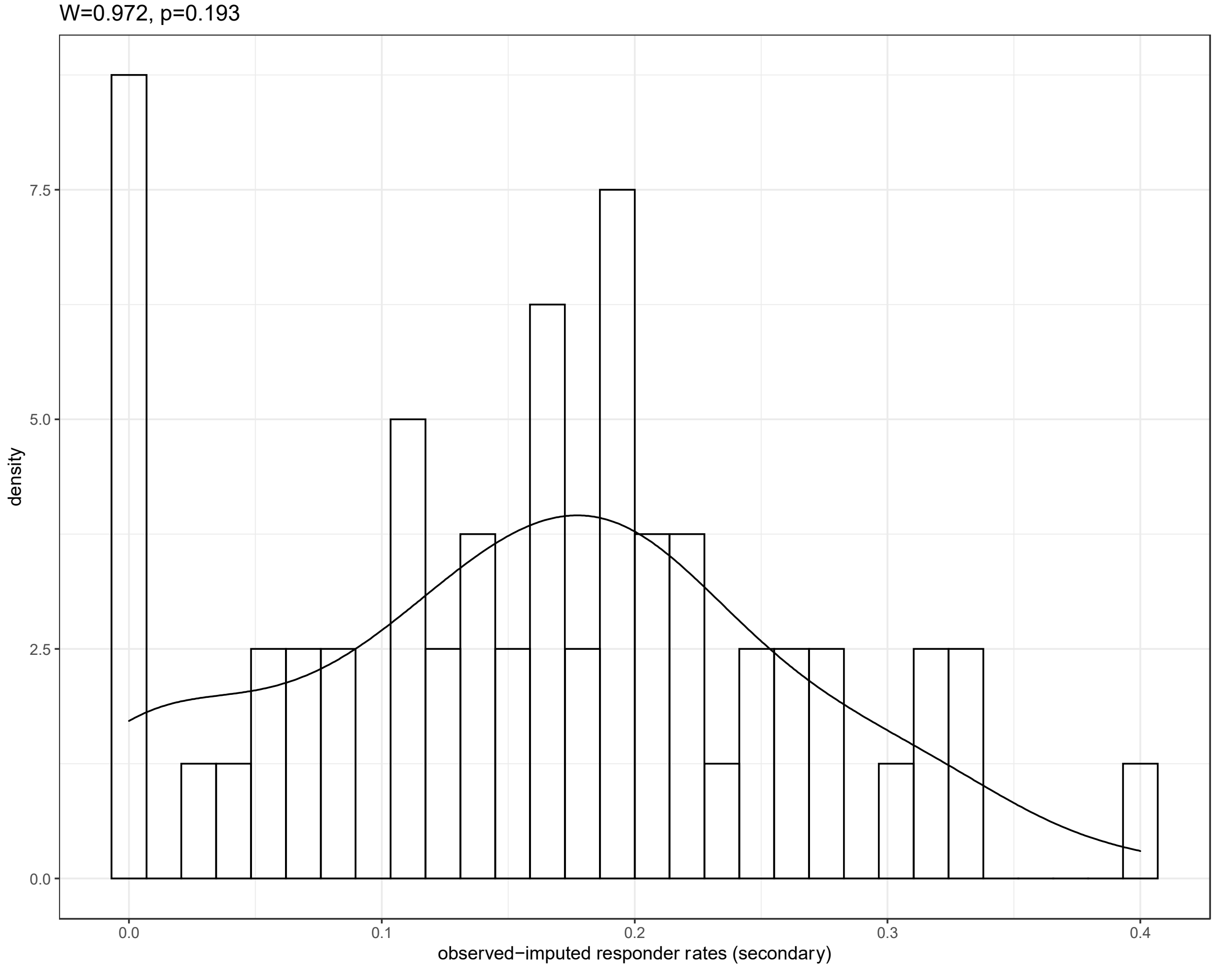
W=0.972, p=0.193
density
7.5
5.0
2.5
0.0
0.0
0.1
0.2
0.3
0.4
observed−imputed responder rates (secondary)

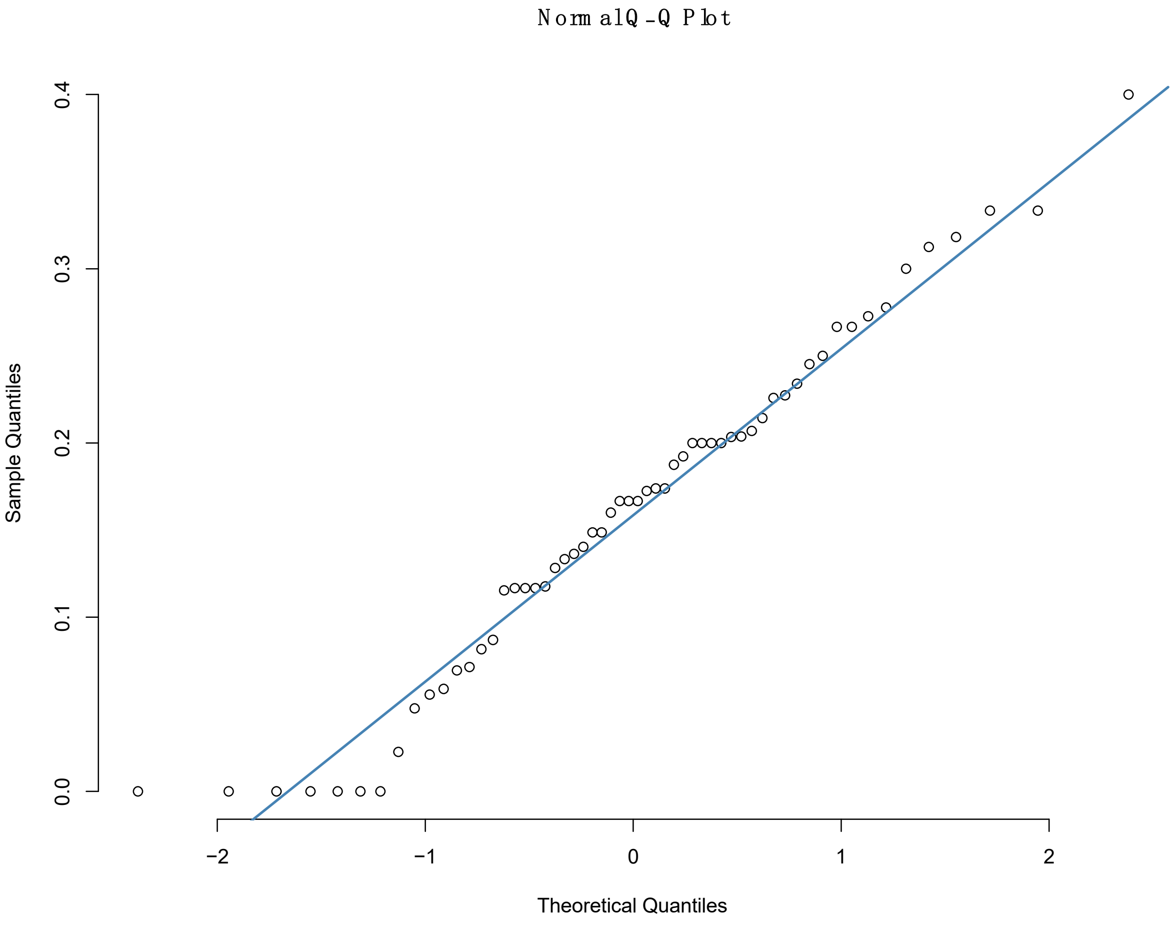
Normal Q-Q Plot
Sample Quantiles
0.0
0.1
0.2
0.3
0.4
Theoretical Quantiles
−2
−1
0
1
2

Figure-S3 Histogram and QQ plot of original-imputed lnOR (primary threshold)

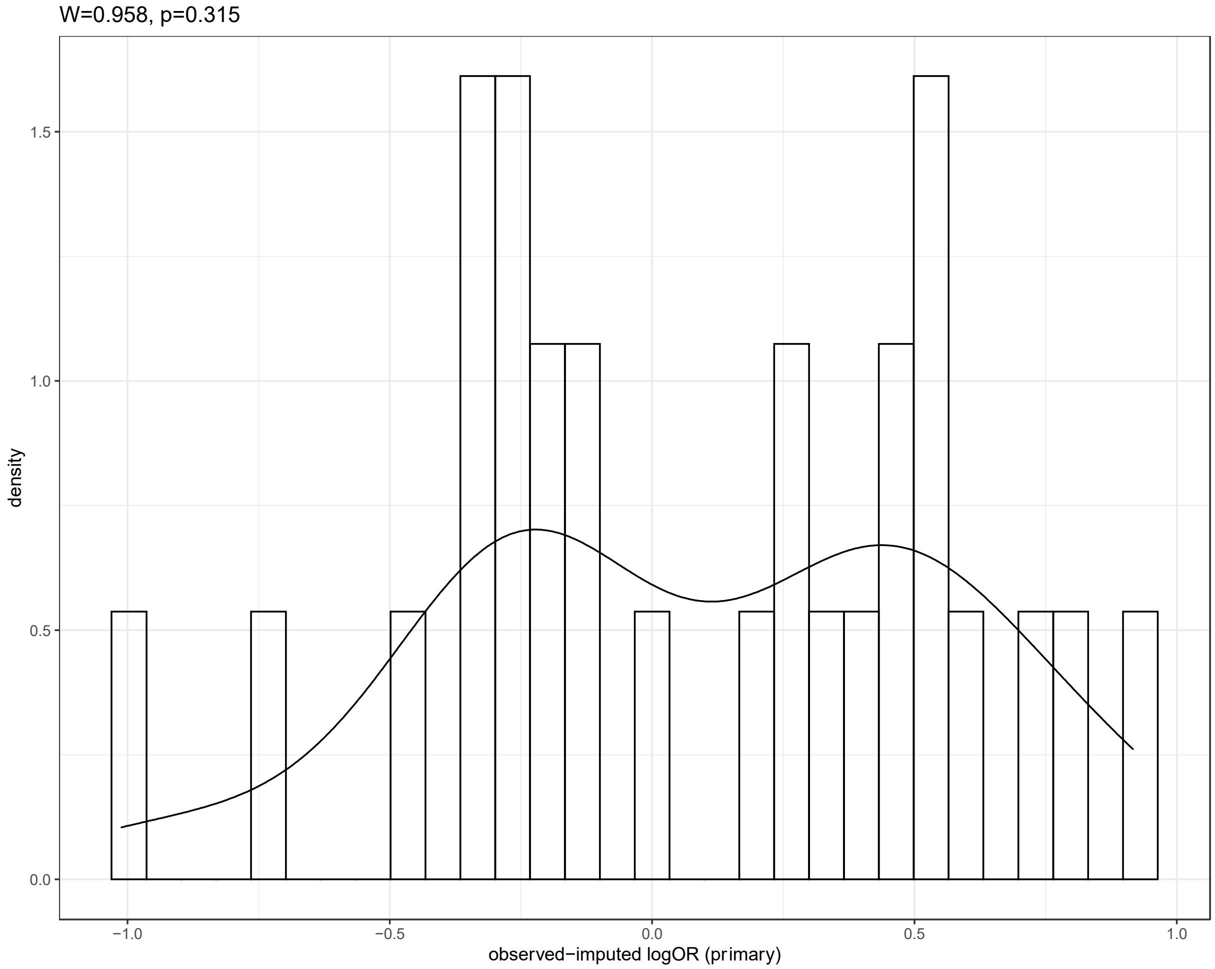

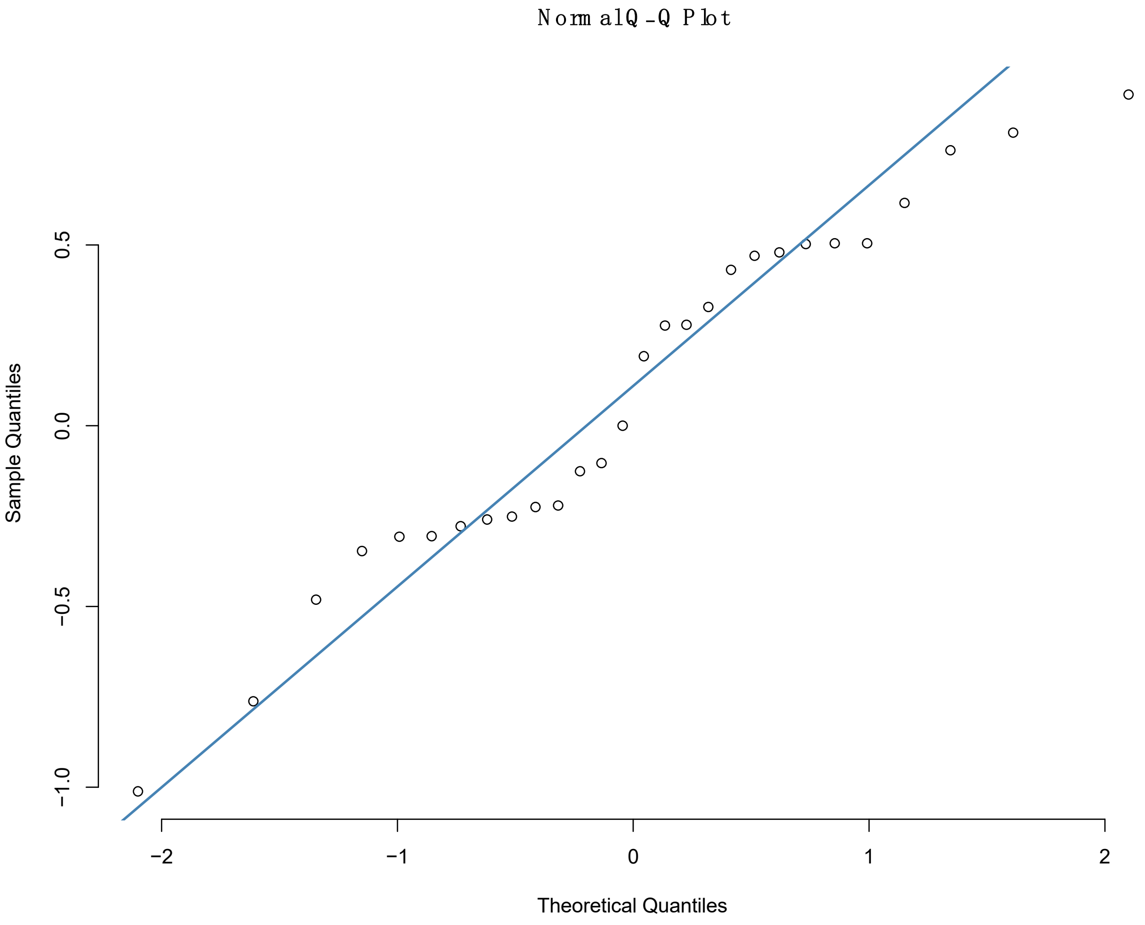
Normal Q-Q Plot
Sample Quantiles
Theoretical Quantiles
0.5
0.0
−0.5
−1.0
−2
−1
0
1
2

Figure-S4 Histogram and QQ plot of original-imputed lnOR (secondary threshold)

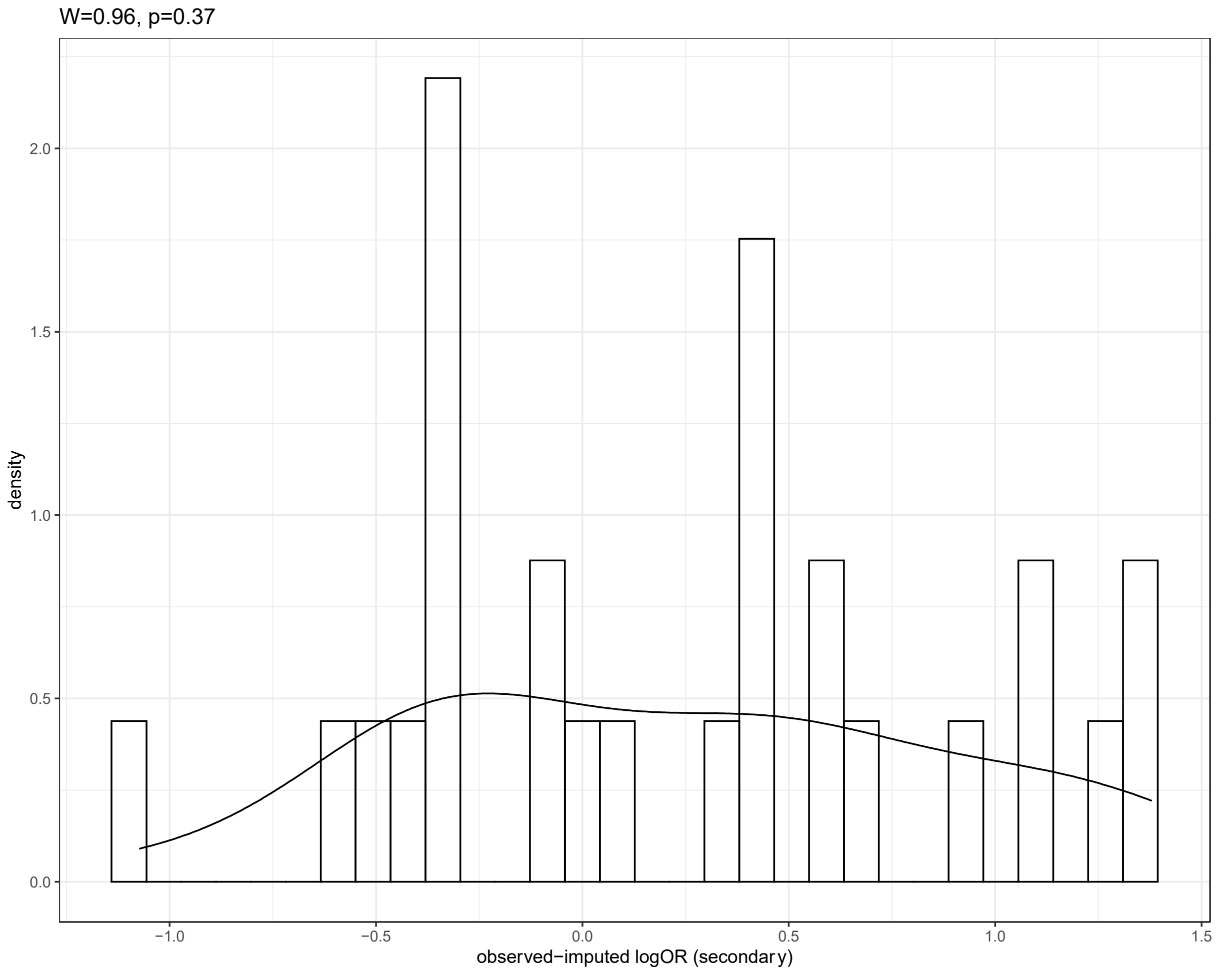

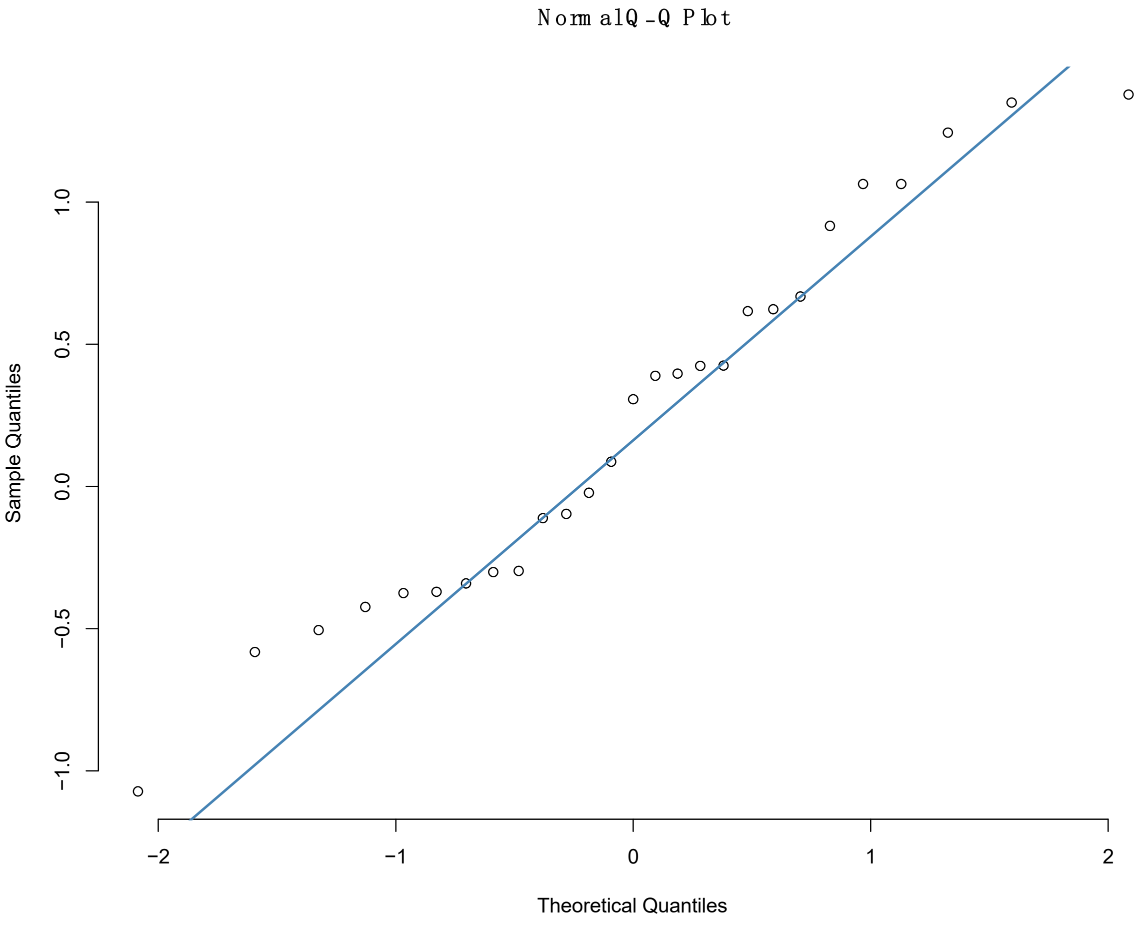

Normal Q-Q Plot
Sample Quantiles
Theoretical Quantiles
−1.0
−0.5
0.0
0.5
1.0
−2
−1
0
1
2

Figure-S5 Investigation of skewness of CGI-I scores

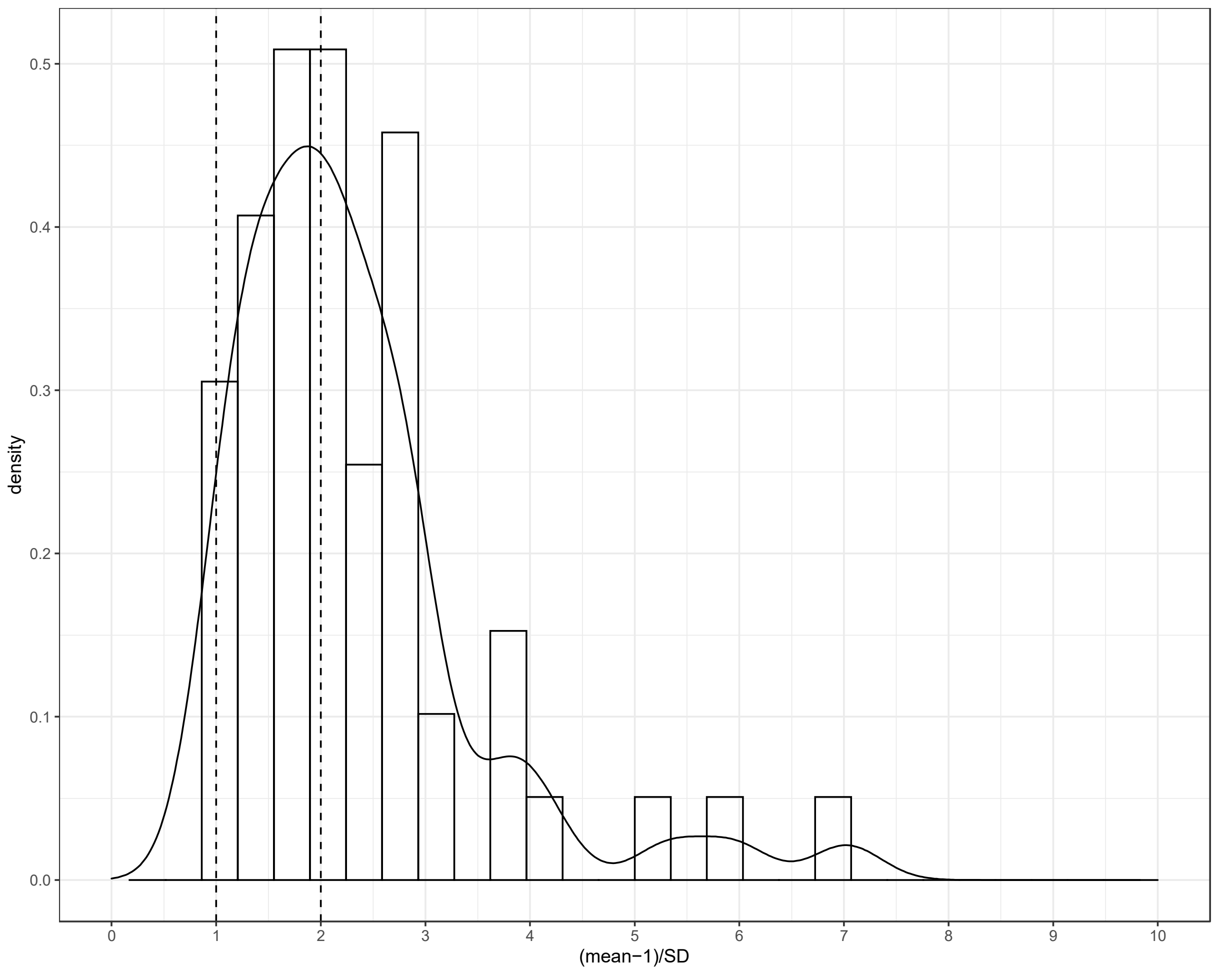

To investigate potential skewness of CGI-I scores from summary data, we followed the Cochrane Handbook and calculated the ratio of mean CGI – 1 (the minimum score of CGI-I) divided by the standard deviation. From the 58 arms, 45 had a ratio below 2 suggesting potential skewness, and 3 had smaller than 1 indicating strong evidence of skewness. The ratio had a median of 2.1 IQR [1.59, 2.75].
